# Inefficacy of *Thymus satureioides* Essential Oil in Cryopreserved Beni Arouss Buck Semen: Post-Thaw Quality Hinges on Antibiotics for Bacterial Control

**DOI:** 10.3390/ani16182845

**Published:** 2026-09-10

**Authors:** Amr Kchikich, Jaber Maataoui, Raoya Khachani, Anass Ben Moula, Nathalie Kirschvink, Bouchra El Amiri, Mehdi El Mokaddem, Youssef Chebli, Samira El Otmani, Said Barrijal, Mouad Chentouf

**Affiliations:** 1 Laboratory of Biotechnological Valorization of Microorganisms, Genomics, and Bioinformatics, Faculty of Sciences and Techniques, University Abdelmalek Essaadi, Tangier 90000, Morocco; s.barrijal@uae.ac.ma; 2 National Institute of Agricultural Research, Regional Center of Agricultural Research of Tangier, Avenue Ennasr, P.O. Box 415 Rabat Principal, Rabat 10090, Morocco; youssef.chebli@inra.ma (Y.C.); samira.elotmani@inra.ma (S.E.O.); 3 Laboratory of Materials, Natural Substances and Environment (LAMSE), Chemistry Department, Faculty of Sciences and Techniques, University Abdelmalek Essaadi, Tangier 90000, Morocco; jaber.maataoui@etu.uae.ac.ma; 4 Research Team in Biotechnology and Biomolecular Engineering, Faculty of Sciences and Techniques, University Abdelmalek Essaadi, Tangier 90000, Morocco; raoya.khachani@etu.uae.ac.ma; 5 Plant, Microbial, Marine, and Precision Agriculture Biotechnology Research Team, Polydisciplinary Faculty of Larache, University Abdelmalek Essaadi, 745 BP, Larache 92004, Morocco; a.benmoula@uae.ac.ma; 6 Department of Medicine, Namur Research Institute for Life Sciences (NARILIS), University of Namur, 5000 Namur, Belgium; nathalie.kirschvink@unamur.be; 7 National Institute for Agricultural Research, Regional Center Agricultural Research of Settat, Avenue Ennasr, P.O. Box 415 Rabat Principal, Rabat 10090, Morocco; bouchra.elamiri@inra.ma; 8 Intelligent Automation and BioMed Genomics Laboratory, Faculty of Sciences and Techniques, University Abdelmalek Essaadi, Tangier 90000, Morocco; elmehdi.elmokaddem@etu.uae.ac.ma

**Keywords:** antibiotics, Beni Arouss bucks, cryoconservation, sperm quality, *Thymus satureioides* essential oil

## Abstract

Growing concerns about antibiotics in semen extenders have driven interest in natural antimicrobial alternatives. The primary objective was to evaluate whether *Thymus satureioides* essential oil (EO) could replace antibiotics in a skim-milk extender while controlling bacterial growth and maintaining the post-thaw quality of Beni Arouss buck semen. Eighty ejaculates from eight bucks were pooled per collection and assigned to 12 treatments: EO at 0–0.05% (*v*/*v*), each with or without penicillin–streptomycin. After cryopreservation, extenders were assessed for oxidative balance, and semen was evaluated for sperm quality, lipid peroxidation, and aerobic bacterial load. Anti-oxidant activity increased and lipid peroxidation in the extender decreased as EO concentration rose whereas antibiotic-containing treatments showed higher lipid peroxidation. In semen, EO at 0.01–0.02% caused no significant changes in motility, viability, morphology, or membrane integrity compared to the control. Antibiotics better preserved sperm quality and reduced bacterial growth, but within 0–0.02% EO they were linked to higher lipid peroxidation. At ≥0.03%, EO showed significant toxicity, sharply reducing sperm quality while bacterial growth correlated negatively with motility, viability, and membrane integrity and positively with abnormalities. These findings suggest that oxidative stress may not have been the primary driver of sperm damage, as much of the measured MDA likely originated from extender lipids. In addition, significant EO × antibiotic interactions for total motility, abnormalities, and bacterial growth indicate that the response to EO depended partly on antibiotic supplementation. Overall, the tested EO concentrations did not reproduce the combined bacterial control and post-thaw sperm preservation observed with antibiotics and therefore did not support replacing antibiotics under these conditions.

## Simple Summary

This research addresses a critical issue in goat farming: how to preserve the semen of Beni Arouss bucks, a local Moroccan goat breed, after freezing and thawing, while controlling both bacterial contamination and oxidative stress (an imbalance that leads to cellular damage). The primary objective was to determine whether *Thymus satureioides* essential oil could replace conventional antibiotics in the semen extender while controlling bacterial growth and preserving sperm quality after freezing and thawing. The tested concentrations did not provide the combined bacterial control and post-thaw sperm preservation observed with antibiotics and therefore did not support antibiotic reduction or replacement. These results underscore that effective bacterial control remains essential for preserving sperm quality, improving animal reproduction, ensuring the conservation of local goat breeds, and guiding research efforts toward the development of safer and more sustainable sperm preservation techniques.

## 1. Introduction

The freezing of sperm allows the preservation of valuable genetics over years and their safe transfer across regions. However, sperm cells are exposed to mechanical and biochemical stresses during freezing and thawing, which decrease post-thaw motility, membrane integrity, mitochondrial function and fertility [1,2,3].

Livestock gene banks are crucial for preserving animal biodiversity by freezing reproductive cells like semen and embryos. These genetic resources are essential for providing alleles to face climate change, and support breed restoration [4].

Artificial insemination at herd level propagates superior genetics, avoiding the transport of live animals, and remains an efficient utilization of frozen semen. Refinements continue to be made in goats, particularly with the use of laparoscopy and the development of devices for intrauterine semen deposition [5]. The progress is based on the consistently high quality of post-thaw semen [5].

Oxidative damage caused by cryopreservation remains a major drawback. ROS are produced due to cold shock, osmotic stress, and mitochondrial imbalance during cooling, freezing, and thawing [1,3]. Biomarkers such as malondialdehyde (MDA; TBARS assay) tend to rise following cryopreservation and are associated with poorer motility, viability, and membrane function; some anti-oxidants are able to partially mitigate this issue [2,6].

A second challenge is bacterial growth, which is introduced during the collection and processing of semen. High bacterial loads are correlated with lower semen quality during storage. The use of antibiotics is increasingly being challenged due to residues, and possible bioeffects on sperm or the female tract [7,8,9]. Alternatives that reduce bacterial loads are being developed to lower the usage of antibiotics without diminishing quality [8,9].

Natural anti-oxidants and antibacterial agents are becoming of interest in this context. Plant EOs, particularly those of the Lamiaceae (thyme, oregano, rosemary) combine redox-modulating and antimicrobial effects. Reviews and experiments across species indicate that appropriately dosed EOs have the potential to enhance motility, membrane function, and oxidative indicators in chilled [10,11] or frozen storage [12]. Such advantages are in line with antibiotic-sparing objectives, and can supplement or, in certain contexts, partly substitute traditional antibiotic cocktails [8].

Importantly, EO effects are dose- and chemotype-dependent. Low, optimized concentrations may protect sperm, but higher doses may be toxic and reduce their motility, disrupting membranes, or functioning as spermicides [10,11,12]. Thus, every EO needs to be carefully dose-optimized and validated in the target species and the extender system [11].

The antimicrobial activity of thymol and carvacrol, the major phenolic monoterpenes in *Thymus satureioides* EO, involves the disruption of bacterial cell membranes by intercalating into the phospholipid bilayer, increasing membrane permeability, and dissipating the proton motive force [13,14]. As anti-oxidants, these phenolic compounds scavenge free radicals by donating hydrogen atoms from their hydroxyl groups, thereby interrupting lipid peroxidation chain reactions and reducing oxidative damage [13,15]. However, these same membrane-active properties can become cytotoxic to eukaryotic cells, including spermatozoa, at higher concentrations, where they may disrupt plasma-membrane integrity, impair mitochondrial function, and alter ionic homeostasis [11,16].

*Thymus satureioides* is a thyme species of particular interest in North Africa because of its thymol- and carvacrol-rich profile, which confers strong antimicrobial and anti-oxidant potential, supporting its consideration in semen preservation [10,15,17]. Indeed, a previous study showed that *Thymus satureioides* EO improved the short-term preservation of Beni Arouss buck semen stored at 4 °C [10]. However, it remains unclear whether these beneficial effects can be maintained under cryopreservation at −196 °C, since deep freezing exposes spermatozoa to much more severe stress than liquid storage, including intracellular ice crystal formation, extreme osmotic changes, and greater oxidative damage [2,3]. Consequently, the results obtained at 4 °C cannot be directly extrapolated to cryopreserved semen, and a dedicated evaluation under cryopreservation conditions is necessary to determine whether the EO retains its protective or antimicrobial properties.

The present study therefore aimed to evaluate the anti-oxidant and antimicrobial effects of different concentrations of *Thymus satureioides* EO, with and without antibiotics, on sperm quality parameters, lipid peroxidation, and bacterial load after freeze–thawing in Beni Arouss bucks, using a factorial design to determine the main and interactive effects of EO and antibiotic supplementation. We hypothesized that, owing to its antimicrobial properties, *Thymus satureioides* essential oil could replace conventional antibiotics in the semen extender without compromising post-thaw sperm quality.

## 2. Materials and Methods

### 2.1. Experimental Design: Semen Collection, Dilution, and Liquid-Nitrogen Storage

This trial was conducted at the Boukhalef experimental station (INRA, Northern Morocco; 35°44′ N, 5°54′ W). Semen was collected using an artificial vagina from eight Beni Arouss bucks once a week for 10 consecutive weeks (80 ejaculates) during the breeding season (November–January). The bucks were housed individually under natural lighting conditions with access to outdoor runs and were fed oat hay and concentrate, with water available ad libitum. Only ejaculates with volume > 1 mL, concentration > 2.5 × 10^9^ sperm/mL, progressive motility > 70%, and >85% normal morphology were pooled within each weekly collection session in order to minimize individual variability and to obtain sufficient volume for all 12 treatments. Each pool served as an independent experimental replicate (*n* = 10 replicates). The pooled semen was divided into twelve equal fractions; a skim-milk extender was supplemented with *Thymus satureioides* EO at 0, 0.01, 0.02, 0.03, 0.04, or 0.05%, each prepared with (CTR+, S1+, S2+, S3+, S4+, S5+; penicillin 50,000 IU and streptomycin 50 mg/100 mL) or without antibiotics (CTR−, S1−, S2−, S3−, S4−, S5−). The EO concentration range (0–0.05%) was selected based on our previous study [10], which identified 0.01% as optimal for chilled semen at 4 °C and showed that 0.05% had toxic effects. At 37 °C the pooled semen was washed in Phosphate-buffered saline (PBS) (1500 g, 10 min) to remove seminal plasma and adjusted by a two-step dilution: first to 8.0 × 10^8^ sperm/mL at 37 °C, then—after 30 min cooling to 4 °C—by adding an equal volume of extender containing 14% glycerol to reach ~4.0 × 10^8^ sperm/mL with 7% glycerol. Aliquots were loaded into 0.25 mL straws, sealed, equilibrated for 2 h at 4 °C, frozen in a controlled-rate freezer (Micro-Digitcool^®^, IMV Technologies, L’Aigle, France) programmed at 3 °C/min from +4 to −5 °C; 25 °C/min from −5 to −110 °C; 35 °C/min from −110 to −150 °C, and plunged into liquid nitrogen (−196 °C). Thawing was performed in a 37 °C water bath for 30 s before post-thaw analyses [12].

### 2.2. Essential Oil Extraction

The fresh aerial parts of *Thymus satureioides* were harvested at the INRA experimental station in Larache, northern Morocco (35°08′ N, 6°08′ W). The material was dried in the shade for 12 days; the leaves were then separated, crushed and hydrodistilled for 3 h using a Clevenger-type apparatus to obtain the EO. The EO was then separated by gravity, collected in amber glass bottles and stored at 4 °C [18]. Its chemical composition had previously been characterized by Gas Chromatography–Mass Spectrometry, confirming a profile dominated by thymol and carvacrol. The anti-oxidant activity of the EO-containing extender was evaluated using the 2,2-diphenyl-1-picrylhydrazyl (DPPH) radical scavenging assay, according to the method described by Kchikich et al., 2022 [12].

### 2.3. Sperm Motility Evaluation

Sperm motion was quantified using computer-assisted sperm analysis (ISAS^®^, Proiser, Valencia, Spain) on a phase-contrast microscope connected to a computer and equipped with a digital camera and a 37 °C heated stage. Before analysis, the samples were adjusted to 30 × 10^6^ sperm/mL, which corresponds to approximately 200 spermatozoa per field. Aliquots of 3 µL were loaded into 20 µm deep chambers on pre-warmed slides to ensure consistent fluid depth and temperature control. For each sample, at least six fields were captured, and total and progressive motility (%) were computed at 10× objective magnification, with frame rate and detection thresholds kept constant throughout the trials [10].

### 2.4. Viability and Abnormality

Sperm viability and morphology were assessed by eosin–nigrosin staining (Minitube^®^, Tiefenbach, Germany) following the procedure outlined by Evans and Maxwell [19]. Briefly, a 3 µL semen drop (4.0 × 10^8^ sperm/mL) was mixed on a pre-warmed slide with 2% eosin and 4% nigrosin (*v*/*v*), spread immediately to form a thin smear, air-dried, and examined at 60×. For each sample, 400 spermatozoa were scored across multiple non-overlapping fields to obtain the percentages of live (unstained) and dead (partially or fully purple-stained) cells, as well as normal and abnormal forms. Morphology was categorized as: head defects (amorphous, pyriform/tapered, elongated, round, acrosomal anomalies, vacuolation), midpiece/neck defects (thick or thin midpiece, bent neck, excess residual cytoplasm, asymmetric neck insertion), and tail defects (double, coiled, short).

### 2.5. Membrane Integrity and Lipid Peroxidation

Sperm plasma-membrane function was assessed with the hypo-osmotic swelling test (HOST): 30 µL semen (4.0 × 10^8^ sperm/mL) was mixed with 300 µL of a 100 mOsm fructose–sodium citrate solution, incubated at 37 °C for 60 min, then 200 spermatozoa were scored at 60× for tail swelling [20].

Post-thaw lipid peroxidation was quantified as thiobarbituric-acid reactive substances (TBARS) in both extender-only samples and semen samples diluted in the extender. Briefly, 0.5 mL of semen or extender sample diluted in (1.8184 g Trishydroxymethyl-aminomethane, 0.9901 g citric acid monohydrate, 50 mL distilled water, pH 7.4) was mixed with 1 mL of the mixture of thiobarbituric acid (15% *w*/*v* trichloroacetic acid, 0.25N hydrochloric acid, 0.375% thiobarbituric acid: *w*/*v* in distilled water) and 1% (*v*/*v*) of butylate hydroxytoluene at 50 mM was added to 500 μL of the sample. Then the mixture was heated at 95 °C for 30 min, cooled on ice, and centrifuged at 3000× *g* for 10 min. The supernatant absorbance was read at 535 nm against reagent blanks. The results were expressed as nmol TBARS per 2.0 × 10^8^ sperm (semen) and nmol TBARS/mL (extenders), using an external MDA standard curve generated from 1,1,3,3-tetraethoxypropane [12]. TBARS measured in semen samples reflected the combined contribution of sperm cells and extender lipids, whereas TBARS measured in extender samples represented oxidation occurring in the extender alone.

### 2.6. Total Aerobic Bacterial Count

Total aerobic bacteria were quantified by spread-plating on Mueller–Hinton agar (Biokar Diagnostics, Allonne, France). Briefly, 1 mL of each semen sample was mixed with sterile PBS (1:10), vortexed, and 100 µL of the dilution was plated in triplicate and incubated aerobically at 37 °C for 48 h; plates with 15–300 colonies were counted and the results expressed as colony-forming units per ml (CFU/mL) [12]. Total aerobic bacterial counts were considered a practical, first-line measure of microbial contamination sufficient to compare the antimicrobial efficacy of EO and antibiotics [7,8].

### 2.7. Statistical Analysis

As the data showed non-normal distributions, percentage data were subjected to an arcsine square root transformation, whereas data expressed in nM or CFU/mL were log_10_-transformed prior to analysis. Normality of model residuals and homogeneity of variances were assessed using the Shapiro–Wilk and Levene’s tests, respectively. After transformation, Levene’s test remained significant (*p* < 0.05) for total motility, progressive motility, viability, abnormality, membrane integrity, semen MDA, and bacterial counts, but was not significant (*p* > 0.05) for anti-oxidant activity and extender MDA. Owing to the balanced experimental design, with 10 replicates per treatment combination, the two-way ANOVA was retained within the GLM framework, considering EO concentration (six levels: 0, 0.01, 0.02, 0.03, 0.04, and 0.05%), antibiotic supplementation (two levels: with and without), and their interaction (EO × antibiotic) as fixed effects. This approach was retained because ANOVA is generally robust to moderate heterogeneity of variances in balanced designs, and this robustness is reinforced here by the equal number of replicates per treatment combination (*n* = 10) [21]. When significant effects were detected, means were compared using Tukey’s HSD test. No additional correction across multiple dependent variables was applied, which is acknowledged as a limitation. Pearson correlation coefficients were calculated using pooled data from the nontoxic EO treatments (0, 0.01, and 0.02% EO, with and without antibiotics; *n* = 60). Because these correlations were derived from pooled data without adjustment for treatment effects, they were considered exploratory associations and were not interpreted as evidence of causation. A *p*-value below 0.05 was considered statistically significant. Data analysis was performed using SAS 9.4 software.

## 3. Results

### 3.1. Anti-Oxidant Activity and Lipid Peroxidation of Extenders

**Figure 1:**
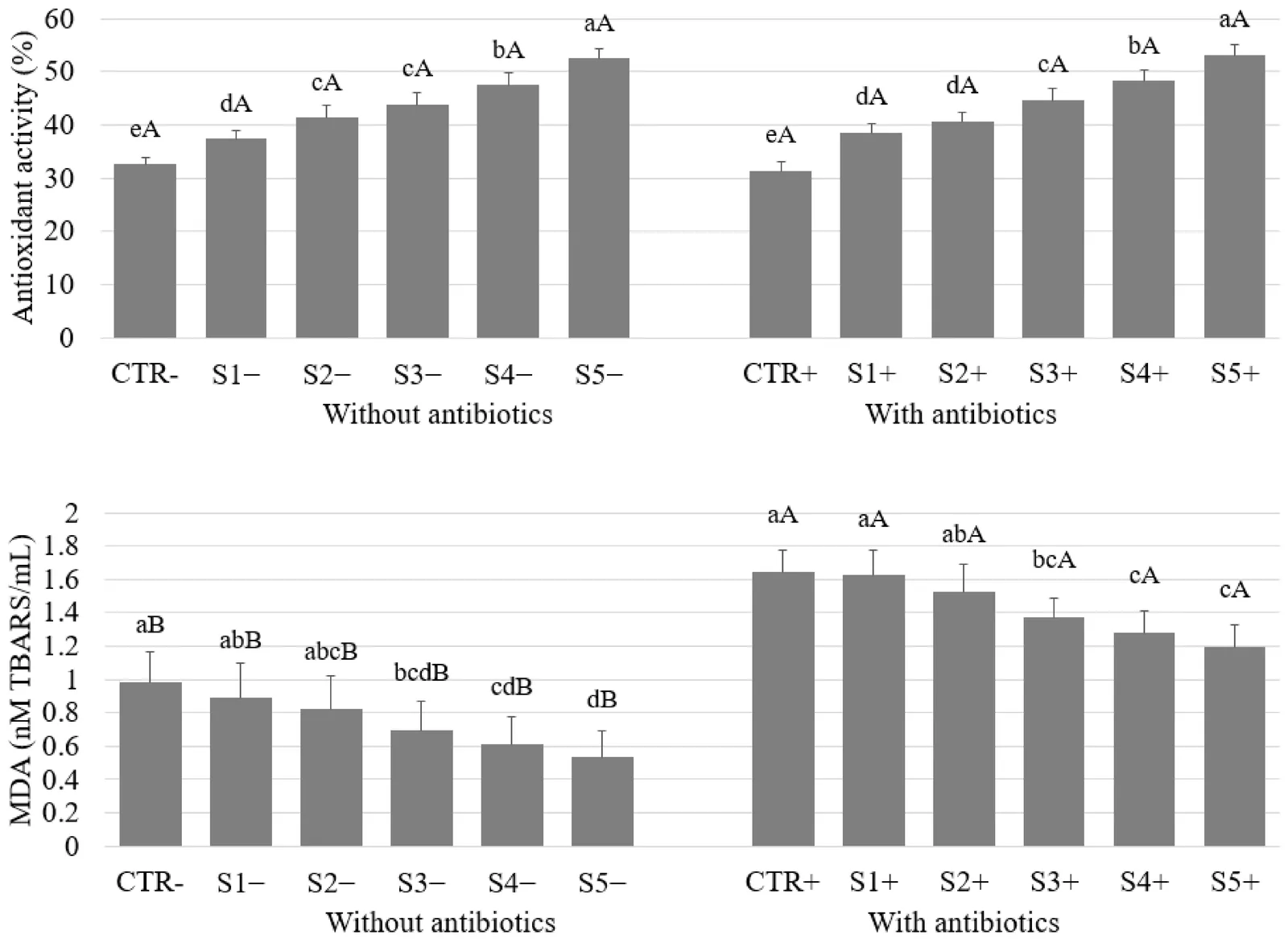
The effect of *Thymus satureioides* essential oil (EO) at different concentrations, with or without antibiotic supplementation, on DPPH free radical scavenging activity (%) and the malondialdehyde concentration (MDA, nM TBARS/mL) of extenders. The lowercase letters (^a, b, c, d, e^) indicate significant differences among EO concentrations within the same antibiotic treatment (*p* < 0.05). The uppercase letters (^A, B^) denote significant differences between antibiotic treatments at a given EO concentration (*p* < 0.05). The data are expressed as mean ± standard deviation from 10 independent pooled semen collections. Statistical significance was set at *p* < 0.05.

The addition of *Thymus satureioides* EO significantly improved the anti-oxidant activity of the diluents in a dose-dependent manner, increasing DPPH scavenging activity from the control group to the S5 group, both in the group with and without antibiotics. At the same time, MDA concentrations decreased progressively as essential oil concentrations increased (*p* < 0.05). However, MDA values were significantly higher in diluents containing antibiotics than in those without antibiotics, for all corresponding essential oil concentrations (Figure 1).

### 3.2. Post-Thaw Total and Progressive Motility

**Figure 2:**
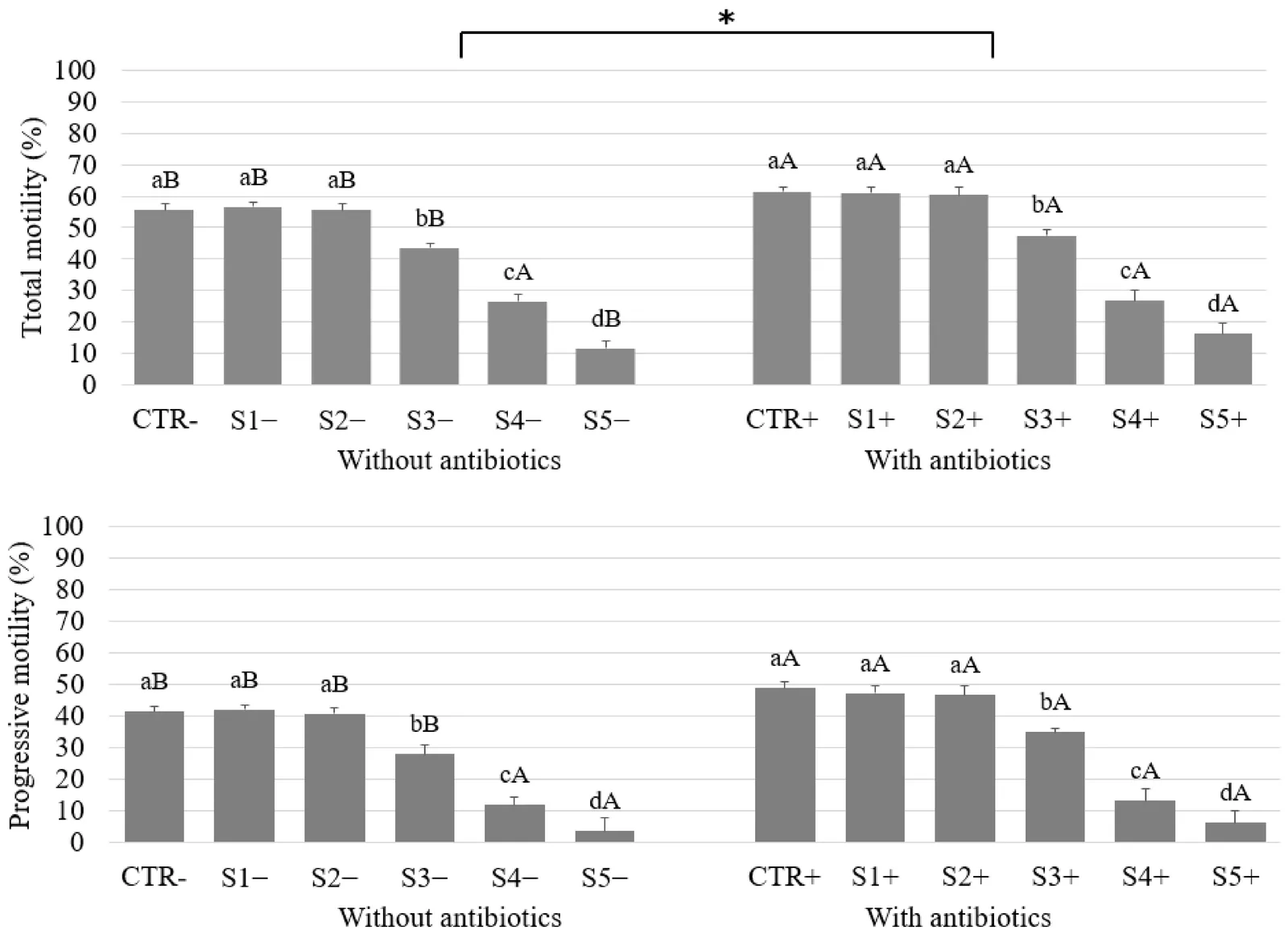
The effect of *Thymus satureioides* essential oil (EO) at different concentrations, with or without antibiotic supplementation, on the post-thaw total motility (%) and progressive motility (%) of buck semen. The lowercase letters (^a, b, c, d^) indicate significant differences among EO concentrations within the same antibiotic treatment (*p* < 0.05). The uppercase letters (^A, B^) denote significant differences between antibiotic treatments at a given EO concentration (*p* < 0.05). The data are expressed as mean ± standard deviation from 10 independent pooled semen collections. Statistical significance was set at *p* < 0.05. * *p* < 0.05 indicates a significant global EO concentration × antibiotic interaction, not a pairwise comparison.

*Thymus satureioides* EO exhibits a dose-dependent reduction in total and progressive motility with a toxicity threshold of ≥0.03%. Total and progressive motility was consistently higher in antibiotic-enriched samples than in those without antibiotics at concentrations up to 0.03%. In both antibiotic and non-antibiotic groups, CTR/S1/S2 (0–0.02%) were statistically similar and showed the highest values, S3 (0.03%) was significantly lower, and S4–S5 (0.04–0.05%) were further reduced. A significant EO × antibiotic interaction was detected for total motility (*, *p* < 0.05) (Figure 2).

### 3.3. Post-Thaw Viability and Abnormality

**Figure 3:**
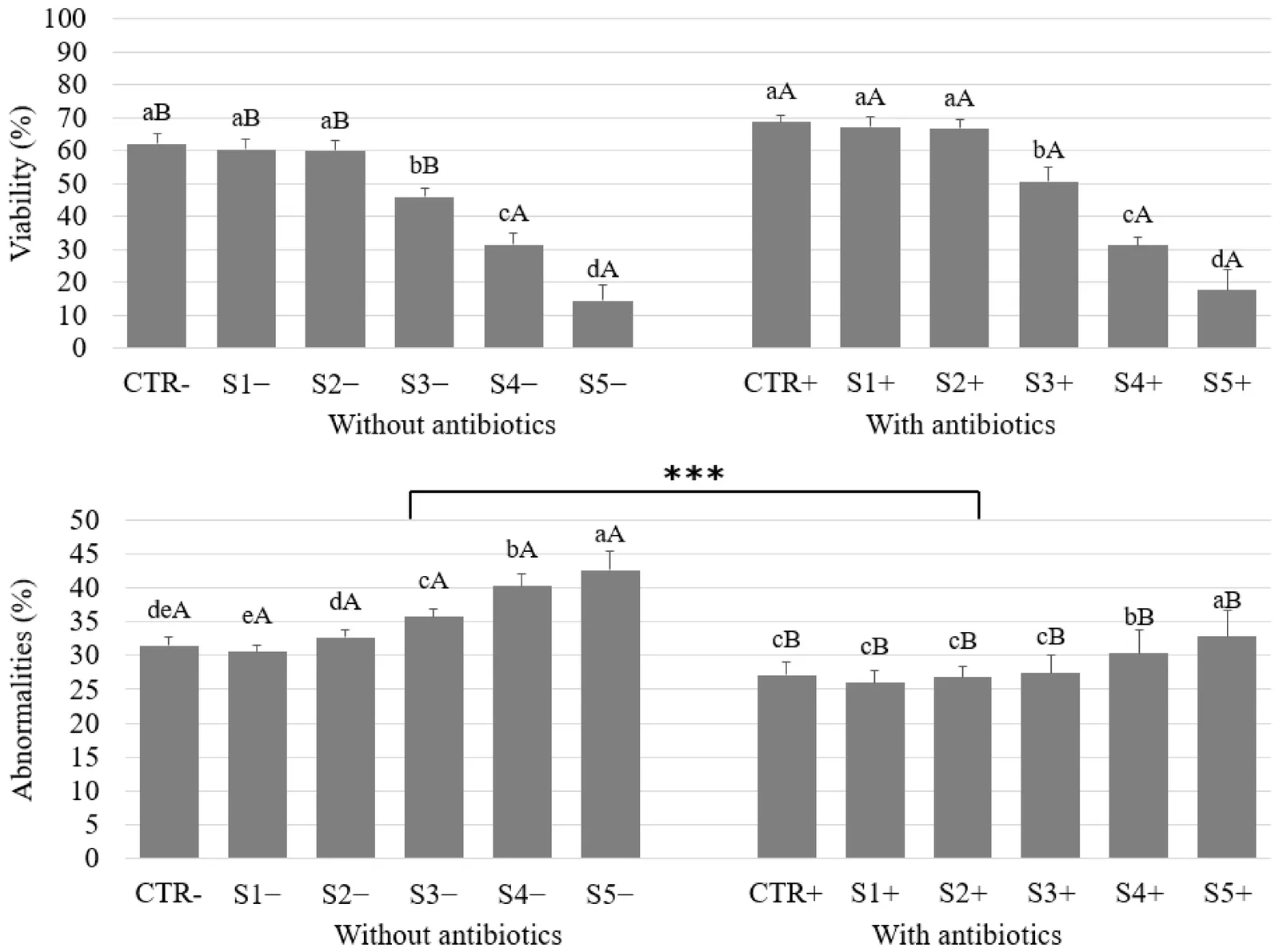
The effect of *Thymus satureioides* essential oil (EO) at different concentrations, with or without antibiotic supplementation, on the post-thaw sperm viability (%) and abnormality morphology (%) of Beni Arouss buck semen. The lowercase letters (^a, b, c, d, e^) indicate significant differences among EO concentrations within the same antibiotic treatment (*p* < 0.05). The uppercase letters (^A, B^) denote significant differences between antibiotic treatments at a given EO concentration (*p* < 0.05). The data are expressed as mean ± standard deviation from 10 independent pooled semen collections. Statistical significance was set at *p* < 0.05. *** *p* < 0.001 indicates a significant global EO concentration × antibiotic interaction, not a pairwise comparison.

The findings show a dose-dependent effect of *Thymus satureioides* EO on buck semen quality, where low concentrations (S1 and S2) maintained high viability and low abnormalities, similar to the control groups. In these groups (CTR, S1 and S2), the samples containing antibiotics consistently showed higher viability and fewer abnormalities than their counterparts without antibiotics. However, higher doses of EO (S3–S5) with and without antibiotics significantly reduced sperm viability and increased morphological abnormalities, demonstrating cytotoxic effects at higher concentrations. A highly significant EO × antibiotic interaction was detected for abnormality (***, *p* < 0.001) (Figure 3).

### 3.4. Post-Thaw Membrane Integrity and Lipid Peroxidation

**Figure 4:**
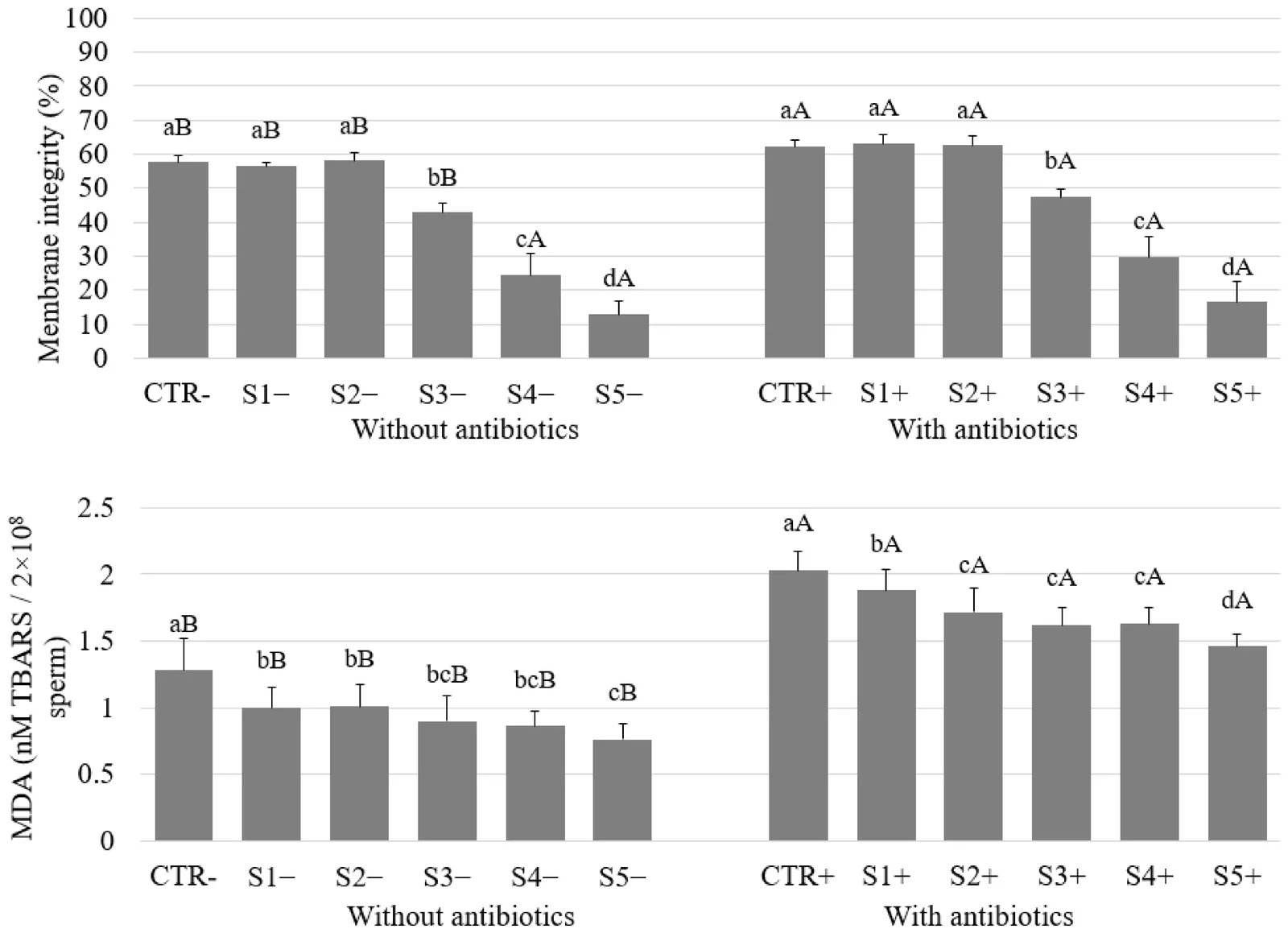
The effect of *Thymus satureioides* essential oil (EO) at different concentrations, with or without antibiotic supplementation, on the post-thaw membrane integrity (%) and lipid peroxidation (MDA, Malondialdehyde nM TBARS/2 × 10^8^ sperm) of Beni Arouss buck semen. The lowercase letters (^a, b, c, d^) indicate significant differences among EO concentrations within the same antibiotic treatment (*p* < 0.05). The uppercase letters (^A, B^) denote significant differences between antibiotic treatments at a given EO concentration (*p* < 0.05). The data are expressed as mean ± standard deviation from 10 independent pooled semen collections. Statistical significance was set at *p* < 0.05.

The findings indicate a clear concentration-dependent reduction in membrane integrity with increasing doses of *Thymus satureioides* EO, in both the presence and absence of antibiotics (*p* < 0.05). While lower EO concentrations (S1, S2) preserved membrane integrity similarly to the control groups, higher concentrations (S3–S5) had a significant negative effect, with the lowest values observed at S5. Antibiotic supplementation significantly preserved membrane stability at 0–0.03% EO concentrations. On the other hand, lipid peroxidation levels (measured by MDA concentration) decreased progressively with increasing EO concentration in the antibiotic-free samples. However, in the presence of antibiotics, MDA levels remained elevated in all groups, including the controls, and only declined significantly at the highest EO dose (S5+) (Figure 4). The measured MDA values represent the combined contribution of sperm and the skimmed milk extender, which already contains a considerable amount of MDA as shown in Figure 1.

### 3.5. Post-Thaw Total Aerobic Bacterial Count

**Figure 5:**
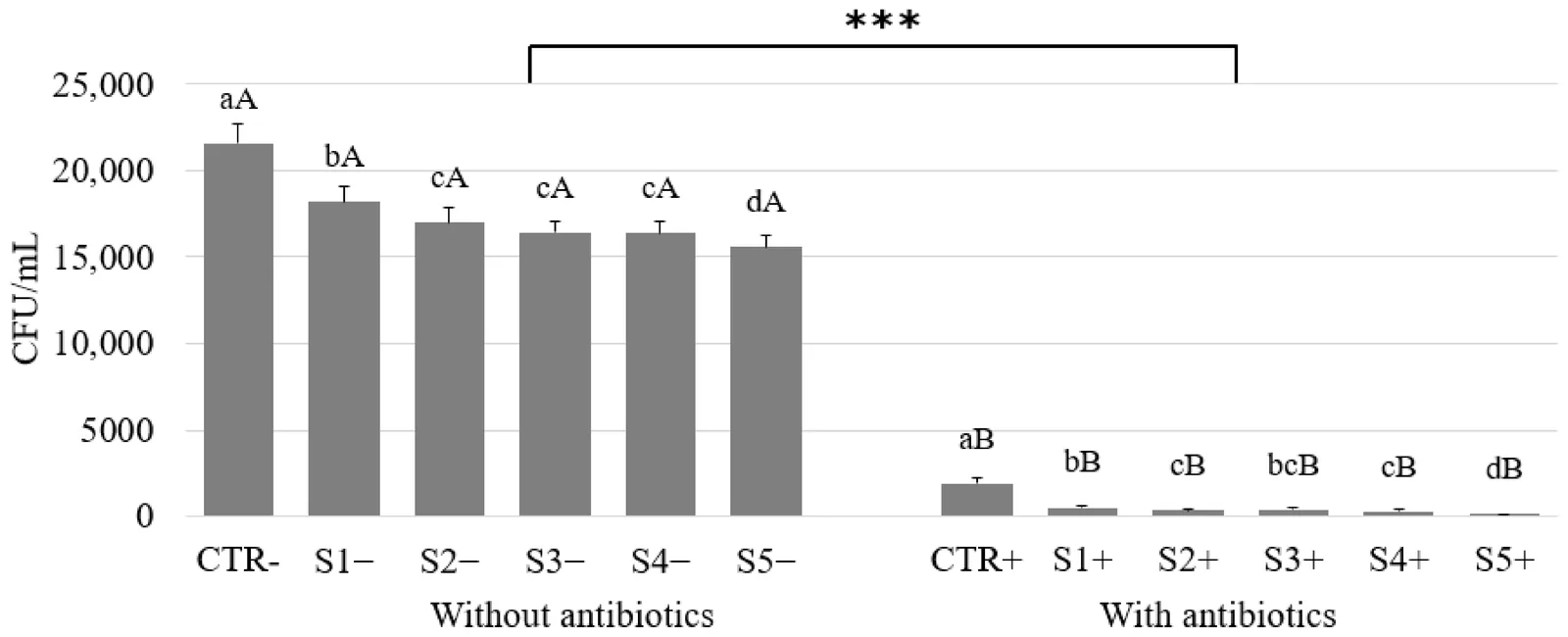
The effect of *Thymus satureioides* essential oil (EO) at different concentrations, with or without antibiotic supplementation, on the post-thaw total aerobic bacterial load (CFU/mL) of buck semen. The lowercase letters (^a, b, c, d^) indicate significant differences among EO concentrations within the same antibiotic treatment (*p* < 0.05). The uppercase letters (^A, B^) denote significant differences between antibiotic treatments at a given EO concentration (*p* < 0.05). The data are expressed as mean ± standard deviation from 10 independent pooled semen collections. Statistical significance was set at *p* < 0.05. *** *p* < 0.001 indicates a significant EO × antibiotic interaction.

The total aerobic counts were significantly higher in all treatments without antibiotics, with the highest value observed in CTR−, followed by a gradual decrease from S1− to S5−. In the antibiotic group, CFU values were drastically reduced across all treatments, with minimal differences between concentrations. A significant effect of antibiotics was observed at all corresponding EO levels (*p* < 0.05). Among the samples without antibiotic, S1− and S2− showed moderate reductions compared to CTR−, while S2−, S3− and S4− were statistically similar, and S5− had the lowest CFU. A highly significant EO × antibiotic interaction was detected for bacterial growth (Figure 5).

### 3.6. Correlation Between Semen Quality Parameters Lipid Peroxidation and Bacterial Growth

The correlation analysis revealed strong inter-relationships among the semen quality parameters lipid peroxidation and bacterial growth (Table 1). Total motility and progressive motility were highly positively correlated (r = 0.99 and 0.96, *p* < 0.001), and both showed strong positive correlations with viability (r = 0.97 and 0.96, respectively) and membrane integrity (r = 0.96 and 0.94, *p* < 0.001). Conversely, abnormality was negatively correlated with these functional parameters, particularly with progressive motility (r = −0.47, *p* < 0.01) and total motility (r = −0.44, *p* < 0.01). Lipid peroxidation showed strong negative correlations with total motility (r = −0.93), progressive motility (r = −0.95), viability (r = −0.96), and membrane integrity (r = −0.91), all at *p* < 0.001, while it was positively correlated with sperm abnormalities (r = 0.56, *p* < 0.001). Importantly, bacterial growth was negatively correlated with all semen quality markers, most strongly with progressive motility (r = −0.78) and lipid peroxidation (r = −0.80), both at *p* < 0.001, and positively correlated with abnormalities (r = 0.85, *p* < 0.001) (Table 1).

## 4. Discussion

Freezing and thawing procedures expose spermatozoa to mechanical, osmotic and oxidative stress, which compromises their motility, membrane integrity and mitochondrial activity [2,3,22], while bacteriospermia further exacerbates these effects through the release of toxins and oxidative spikes [7,8]. In this context of oxidative and microbial stress, *Thymus satureioides* EO, rich in thymol and carvacrol, has powerful anti-oxidant and antibacterial properties [10,13,14,17]. At low concentrations, the EO improves redox balance and limits bacterial growth, while at higher doses it becomes spermatotoxic [16].

In this study, analysis of diluents enriched with EO alone (Figure 1) showed a dose-dependent increase in anti-oxidant activity accompanied by a decrease in MDA concentration, reflecting a lower oxidative state of the storage medium. These MDA values correspond exclusively to the skimmed milk extender, which naturally contains oxidizable lipid and protein fractions that are likely to oxidize spontaneously during storage [23,24].

*Thymus satureioides* EO has been shown to have a narrow operational range (0.01 to 0.02%) on total and progressive motility. Within this range, regardless of the presence or absence of antibiotics, the modest anti-oxidant intake at these low doses of *Thymus satureioides* EO is unlikely to overcome this damage compared to the control (*p* > 0.05). A significant EO × antibiotic interaction was observed for total motility (*p* < 0.05), indicating that the motility response to EO varied according to the presence or absence of antibiotics. Similar findings have been reported in ram and buck semen; low concentrations of oregano or rosemary EO maintained motility at 4 °C but showed limited or no benefit after freeze–thawing [12,25].

The lower CFU counts observed in antibiotic-containing treatments coincided with better preservation of post-thaw total and progressive motility. This association is consistent with previous evidence that bacteriospermia may impair sperm movement through agglutination and through bacterial adhesion or bacterial products that can increase oxidative stress and disturb mitochondrial energy production [7,26]. Although agglutination, ROS production, mitochondrial function, and ATP availability were not directly assessed in the present study, these mechanisms provide a plausible explanation for the negative correlations observed between bacterial load and sperm motility. In contrast, EO concentrations ≥ 0.03% caused a dose-dependent decline in motility, with progressive motility being affected more markedly than total motility, despite the reduction in bacterial counts. This divergence suggests that, at high concentrations, the detrimental effects of EO on spermatozoa outweighed its antibacterial activity. Thymol and carvacrol are membrane-active phenolic compounds, and their interaction with the sperm plasma membrane at elevated concentrations could secondarily affect mitochondrial function and flagellar movement [11,13,16]. In bull spermatozoa, similar dose-dependent toxicity has been reported with oregano EO at concentrations exceeding 0.025%, manifesting as progressive decline in motility and mitochondrial membrane potential [16].

No significant differences were observed in terms of viability or normal morphology between the control group and the groups treated with 0.01% or 0.02% *Thymus satureioides* EO with and without antibiotics. However, at concentrations ≥ 0.03%, a gradual cytotoxic profile was observed, with a decrease in live cell counts and a parallel increase in abnormalities in the head, mid/neck and tail regions, indicating concentration-dependent sperm toxicity. The parallel deterioration in viability and morphology suggests that, at high concentrations, interactions between hydrophobic EO constituents and sperm membranes may have aggravated the structural damage associated with freezing and thawing [2,3,25]. Such membrane and cytoskeletal instability could account for the variety of abnormalities observed across the head, midpiece/neck, and tail regions [2,27]. Similar concentration-dependent morphological damage has been reported with other EOs in ram and goat semen [16,25], supporting the existence of a narrow tolerated concentration range. The highly significant EO × antibiotic interaction observed for abnormalities further supports that the morphological response to increasing EO concentration differed markedly according to antibiotic supplementation.

The presence of bacteriospermia may contribute to another axis of damage: bacterial adhesion and lipopolysaccharides/exotoxins have been reported to increase ROS and Ca^2+^ dysregulation, promoting membrane damage and abnormal morphology. Consequently, a reduction in CFU improves viability and normal morphology [7,26]. The beneficial effects observed at low doses were mainly due to antibiotics, which reduced bacterial load, an effect that can be achieved by adding antibiotics directly to the skim-milk extender without EO supplementation [7,8]. At concentrations ≥ 0.03%, the membrane and structural damage induced by EO outweighed any bactericidal or bacteriostatic benefit.

At concentrations of 0.01% and 0.02%, with and without antibiotics, no significant difference was observed compared to the control in terms of membrane integrity. However, at concentrations ≥ 0.03%, EO caused a marked decrease in membrane integrity values, probably due to the progressive intercalation of hydrophobic phenolic compounds into the lipid bilayer, resulting in surfactant and protonophoric effects that disrupted transmembrane potentials and membrane protein functions [13,28].

The addition of antibiotics showed a significant improvement compared to the control group in terms of preserving membrane integrity. In this context, studies have shown that the addition of penicillin–streptomycin indirectly protects the sperm membrane by limiting the survival of opportunistic bacteria such as *Escherichia coli* and *Staphylococcus* spp., which weaken the lipid bilayer of the plasma membrane and promote structural damage to the head, midpiece, and flagellum rather than acting as direct membrane cryoprotectants [29,30].

In the antibiotic-treated group, the systematically higher MDA levels observed in diluents with and without sperm should not be considered sperm-specific, since TBARS values may include contributions from both spermatozoa and the skim-milk-based diluent. One possible explanation is that, although bactericidal activity reduces the number of viable bacteria, it may also induce oxidative bursts and the release of bacterial products in contaminated samples, thereby increasing ROS in the medium and promoting lipid peroxidation [8,26,31]. In our study, however, these higher MDA values were observed despite lower bacterial loads and better semen quality in antibiotic-treated samples, suggesting that bacterial control was more closely associated with semen preservation than MDA levels alone. This interpretation is further supported by the strong positive correlations observed between MDA and total and progressive motility, viability, and membrane integrity, which, in light of the mixed sperm and diluent derived origin of the TBARS signal, are contrary to the inverse relationship generally expected under oxidative damage conditions [32,33]. Likewise, although the anti-oxidant activity of *Thymus satureioides* EO increased from 0 to 0.02%, this did not translate into improved sperm preservation compared with the corresponding controls. Alternatively, a direct pro-oxidant effect of the penicillin–streptomycin mixture and/or its interaction with the skim-milk extender cannot be excluded, since several clinically used antibiotics can modulate redox homeostasis and promote ROS-related oxidative stress in nonbacterial systems [34]. These two mechanisms are not mutually exclusive and may operate in parallel. Distinguishing between them would require further studies using cell-free or bacteria-free extender systems. Overall, these observations suggest that, under the conditions of the present study, oxidative stress was unlikely to be the sole determinant of post-thaw sperm damage, and this hypothesis should be further examined using sperm-specific oxidative stress markers.

The total number of aerobic bacteria was biologically meaningful because it was inversely associated with total and progressive motility, viability, and membrane integrity, and positively associated with abnormal morphology, in agreement with the reported harmful effects of bacteriospermia on sperm function [35,36]. The greatest decrease in CFU count and overall improvement in quality were observed in the antibiotic-treated group, while a low dose of *Thymus satureioides* EO (0.01% and 0.02%) moderately reduced bacterial growth in both the antibiotic-treated and antibiotic-free groups. The highly significant EO × antibiotic interaction for bacterial growth (*p* < 0.001) indicates that the antibacterial response to EO depended on antibiotic supplementation, with 0.02% EO further reducing bacterial counts in antibiotic-containing extenders. However, this additional reduction in bacterial load did not translate into a consistent improvement in post-thaw sperm quality parameters. Overall, these findings support a strong association between bacterial load and post-thaw sperm quality (*p* < 0.05).

Our findings are consistent with a growing body of evidence across species indicating that plant EOs have a narrow therapeutic window in semen preservation. In ram semen, rosemary extract at 0.01–0.02% preserved post-thaw motility, but higher concentrations (≥0.05%) were spermicidal [25]. In poultry, thymol-based additives at low doses improved sperm viability in liquid storage, although cryopreservation data remain limited [16]. In bull semen, carvacrol at 25–50 µg/mL improved post-thaw motility in some studies, whereas higher doses reduced membrane integrity [16]. Taken together, our results indicate that the initial hypothesis was not supported under the cryopreservation conditions tested, since low EO concentrations were tolerated but did not reproduce the protective effect of antibiotics on post-thaw sperm quality and bacterial control. Collectively, these findings underscore the importance of species-specific and extender-specific dose optimization and caution against extrapolating results across cryopreservation and liquid storage conditions.

## 5. Conclusions

Under the conditions of this study, low concentrations of *Thymus satureioides* EO (0.01–0.02%) in a skim-milk-based extender were tolerable after freeze–thawing of Beni Arouss buck semen, producing no significant differences in motility, viability, morphology, or membrane integrity compared to the control. At concentrations ≥ 0.03%, the EO caused dose-dependent cytotoxicity and spermicidal effects. The EO reduced total aerobic bacterial counts in some extenders; however, antibiotic-containing treatments achieved markedly stronger bacterial control and generally better preservation of post-thaw sperm quality. Consequently, the tested EO concentrations did not provide the combined antibacterial efficacy and sperm preservation required to support the replacement of antibiotics. From an oxidative perspective, EO increased DPPH radical scavenging activity and progressively reduced extender MDA/TBARS; however, these changes were not accompanied by improved post-thaw sperm quality. Although MDA/TBARS was significantly associated with sperm quality parameters, its interpretation as a sperm-specific marker of oxidative damage was limited because the semen measurements reflected contributions from both spermatozoa and the skim-milk extender. Further studies using sperm-specific oxidative stress markers are therefore needed to clarify the contribution of oxidative damage to sperm cryoinjury.

## Figures and Tables

**Table 1:** Correlation coefficients between semen quality parameters lipid peroxidation and bacterial growth.

	Total Motility	Progressive Motility	Viability	Abnormality	Membrane Integrity	Lipid Peroxidation	Bacterial Growth
Total motility	-	0.99 ***	0.97 ***	−0.44 **	0.96 ***	−0.93 ***	−0.76 ***
Progressive motility		-	0.96 ***	−0.47 **	0.94 ***	−0.95 ***	−0.78 ***
Viability			-	−0.37 **	0.95 ***	−0.96 ***	−0.69 ***
Abnormality				-	−0.35 **	0.56 ***	0.85 ***
Membrane integrity					-	−0.91 ***	−0.73 ***
Lipid peroxidation						-	−0.80 ***
Bacterial growth							-

Note: The semen was diluted with 0, 0.01% and 0.02% of *Thymus satureioides* EO with and without antibiotics. The significance level for correlation coefficient analysis was set at ** *p* < 0.01 and *** *p* < 0.001.

## Data Availability

The original contributions presented in this study are included in the article. Further inquiries can be directed to the corresponding authors.

## Abbreviations

The following abbreviations are used in this manuscript:

EO	Essential oil
PBS	Phosphate-buffered saline
DPPH	2,2-diphenyl-1-picrylhydrazyl
CTR+	Skim-milk with antibiotics
S1+	Skim-milk containing antibiotics and supplemented with 0.01% of *Thymus satureioides* EO
S2+	Skim-milk containing antibiotics and supplemented with 0.02% of *Thymus satureioides* EO
S3+	Skim-milk containing antibiotics and supplemented with 0.03% of *Thymus satureioides* EO
S4+	Skim-milk containing antibiotics and supplemented with 0.04% of *Thymus satureioides* EO
S5+	Skim-milk containing antibiotics and supplemented with 0.05% of *Thymus satureioides* EO
CTR−	Skim-milk without antibiotics
S1−	Skim-milk supplemented with 0.01% of *Thymus satureioides* EO
S2−	Skim-milk supplemented with 0.02% of *Thymus satureioides* EO
S3−	Skim-milk supplemented with 0.03% of *Thymus satureioides* EO
S4−	Skim-milk supplemented with 0.04% of *Thymus satureioides* EO
S5−	Skim-milk supplemented with 0.05% of *Thymus satureioides* EO
MDA	Malondialdehyde
HOST	Hypo-osmotic swelling test
TBARS	Thiobarbituric-acid reactive substances
CFU	Colony-forming units
